# Nanoscale alterations in GABA_B_ receptors and GIRK channel organization on the hippocampus of APP/PS1 mice

**DOI:** 10.1186/s13195-022-01078-5

**Published:** 2022-09-21

**Authors:** Alejandro Martín-Belmonte, Carolina Aguado, Rocío Alfaro-Ruiz, Ana Esther Moreno-Martínez, Luis de la Ossa, Ester Aso, Laura Gómez-Acero, Ryuichi Shigemoto, Yugo Fukazawa, Francisco Ciruela, Rafael Luján

**Affiliations:** 1 Synaptic Structure Laboratory, Instituto de Investigación en Discapacidades Neurológicas (IDINE), Department Ciencias Médicas, Facultad de Medicina, Universidad Castilla-La Mancha, Campus Biosanitario, C/ Almansa 14, 02008 Albacete, Spain; 2 Pharmacology Unit, Department of Pathology and Experimental Therapeutics, Faculty of Medicine and Health Sciences, Institute of Neurosciences, University of Barcelona, 08907 L’Hospitalet de Llobregat, Spain; 3 Neuropharmacology and Pain Group, Neuroscience Program, Institut d’Investigació Biomèdica de Bellvitge, IDIBELL, 08907 L’Hospitalet de Llobregat, Spain; 4 Departamento de Sistemas Informáticos, Escuela Superior de Ingeniería Informática, Universidad de Castilla-La Mancha, 02071 Albacete, Spain; 5 Institute of Science and Technology Austria (ISTA), Am Campus 1, A-3400 Klosterneuburg, Austria; 6 Division of Brain Structure and Function, Faculty of Medical Science, University of Fukui, Fukui, Japan; 7 Life Science Innovation Center, University of Fukui, Fukui, Japan

## Abstract

Alzheimer’s disease (AD) is characterized by a reorganization of brain activity determining network hyperexcitability and loss of synaptic plasticity. Precisely, a dysfunction in metabotropic GABA_B_ receptor signalling through G protein-gated inwardly rectifying K^+^ (GIRK or Kir3) channels on the hippocampus has been postulated. Thus, we determined the impact of amyloid-β (Aβ) pathology in GIRK channel density, subcellular distribution, and its association with GABA_B_ receptors in hippocampal CA1 pyramidal neurons from the APP/PS1 mouse model using quantitative SDS-digested freeze-fracture replica labelling (SDS-FRL) and proximity ligation in situ assay (P-LISA). In wild type mice, single SDS-FRL detection revealed a similar dendritic gradient for GIRK1 and GIRK2 in CA1 pyramidal cells, with higher densities in spines, and GIRK3 showed a lower and uniform distribution. Double SDS-FRL showed a co-clustering of GIRK2 and GIRK1 in post- and presynaptic compartments, but not for GIRK2 and GIRK3. Likewise, double GABA_B1_ and GIRK2 SDS-FRL detection displayed a high degree of co-clustering in nanodomains (40–50 nm) mostly in spines and axon terminals. In APP/PS1 mice, the density of GIRK2 and GIRK1, but not for GIRK3, was significantly reduced along the neuronal surface of CA1 pyramidal cells and in axon terminals contacting them. Importantly, GABA_B1_ and GIRK2 co-clustering was not present in APP/PS1 mice. Similarly, P-LISA experiments revealed a significant reduction in GABA_B1_ and GIRK2 interaction on the hippocampus of this animal model. Overall, our results provide compelling evidence showing a significant reduction on the cell surface density of pre- and postsynaptic GIRK1 and GIRK2, but not GIRK3, and a decline in GABA_B_ receptors and GIRK2 channels co-clustering in hippocampal pyramidal neurons from APP/PS1 mice, thus suggesting that a disruption in the GABA_B_ receptor–GIRK channel membrane assembly causes dysregulation in the GABA_B_ signalling via GIRK channels in this AD animal model.

## Introduction

G protein-gated inwardly rectifying K^+^ (GIRK) channels constitute a major effector system of G protein-coupled receptor (GPCR) signalling through G_αi/o_ proteins, thus modulating neuron excitability and spike firing via slow inhibitory postsynaptic potentials [1, 2]. In addition, pharmacological manipulations and genetic mouse models have related GIRK channels to synaptic plasticity and cognitive behaviour [3, 4]. Interestingly, a main GPCR activating GIRK channel is the metabotropic γ-aminobutyric acid type B (GABA_B_) receptor [5], which is involved in many physiological and pathological conditions [6], including Alzheimer’s disease (AD) [7–9]. Indeed, there is a growing body of evidence pointing towards the existence of a dysregulation in the signalling operated by GABA_B_ receptors and GIRK channels in AD [9–11].

Molecular cloning techniques have identified four GIRK channel subunits designated as GIRK1, GIRK2, GIRK3, and GIRK4 [1]. Although all four subunits are expressed in the brain [12], neural GIRK channels are homo- or heterotetrameric complexes formed by the association of GIRK1, GIRK2, and GIRK3 subunits [1, 2, 12, 13]. This subunit combination depends on the neuronal type and the subcellular compartment [14–17]. Interestingly, GIRK1-3 subunits are expressed on the hippocampus [12], one of the earliest and most affected brain regions in AD [18]. Importantly, preclinical models recreating certain AD neuropathological hallmarks have correlated increased density of amyloid-β peptide (Aβ) with a downregulation in the gene expression of hippocampal GIRK channels and with a reduction in GIRK conductance in pyramidal cells [10, 19]. Furthermore, GIRK channel internalization in CA1 pyramidal neurons has been also demonstrated in different AD mouse models [20].

On the hippocampus, GIRK channels preferentially localize to the extrasynaptic plasma membrane of spines, dendrites, and axon terminals [16, 21, 22], where they colocalize with GABA_B_ receptors [16, 22, 23]. The view that GABA_B_ receptors and GIRK channels form stable macromolecular membrane assemblies (MMA) [24] to ensure a specific and fast signalling under physiological conditions is supported by experimental evidence obtained both in native and heterologous systems [22, 25–27]. However, it remains unclear whether these GABA_B_ receptor–GIRK channel MMA undergo alteration upon pathological conditions. In spite that we have recently demonstrated a significant reduction in the plasma membrane of both GABA_B_ receptors [9] and GIRK2 channels [20] in the APP/PS1 mouse model, the effect of Aβ pathology in GIRK1 and/or GIRK3 density and subcellular distribution still is unknown. To this end, here we aimed to provide a nanoscale view of the organization of GIRK channel subunits, their spatial relationship with GABA_B_ receptors, and the formation of GABA_B1_/GIRK2 oligomeric complexes (i.e. MMA) in the hippocampus of APP/PS1 mice by using quantitative immunoelectron microscopy and P-LISA techniques.

## Material and methods

### Animals

Male APP/PS1 mice (RRID:IMSR_MMRRC:034832) were obtained from the Jackson Laboratory (https://www.jax.org/strain/005864) and expressed Mo/Hu APP695swe construct in conjunction with the exon-9-deleted variant of human presenilin 1 [Tg(APPswe,PSEN1dE9)85Dbo/Mmjax] [28, 29]. The “control” wild type (WT) mice were age-matched littermates without the transgene. For analysis, we selected animals of 12 months of age, characterized by memory deficits with severe synapse loss and widespread Aβ deposition [30, 31]. For each genotype, four mice were used for SDS-digested freeze-fracture replica labelling (SDS-FRL) and four mice were used for P-LISA. Mice for SDS-FRL and P-LISA were maintained at the Animal House Facility of the University of Castilla-La Mancha (Albacete, Spain) and the University of Barcelona (Barcelona, Spain), respectively, in cages of 2 or more mice, on a 12-h light/12-h dark cycle at 24°C and received food and water ad libitum. Care and handling of animals prior to and during experimental procedures were in accordance with Spanish (RD 53/2013) and European Union regulations (2010/63/UE), and all protocols and methodologies were approved by the Animal Care and Use Committees of the two Universities.

For SDS-FRL experiments, animals were anesthetized with sodium pentobarbital (50 mg/kg, i.p.) and perfused transcardially with 25 mM PBS for 1 min, followed by perfusion with 2% paraformaldehyde in 0.1 M phosphate buffer (PB) for 12 min. After perfusion, brains were removed, and the hippocampi were dissected and cut into sagittal slices (130 μm) using a Microslicer (Dosaka, Kyoto, Japan) in 0.1 M PB. For P-LISA, mice were anesthetized and perfused transcardially with 25 mM PBS for 1 min, followed by perfusion with 4% paraformaldehyde in phosphate-buffered saline (PBS; 8.07 mM Na_2_HPO_4_, 1.47 mM KH_2_PO_4_, 137 mM NaCl, 0.27 mM KCl, pH 7.2). Coronal sections (60 μm) were processed using a vibratome (Leica Lasertechnik GmbH, Heidelberg, Germany). Slices were collected in Walter’s anti-freezing solution (30% glycerol, 30% ethylene glycol in PBS, pH 7.2) and kept at −20°C until processing.

### Antibodies and chemicals

The primary antibodies used were the following: rabbit anti-GABA_B1_ polyclonal (B17, aa. 525–539 of mouse GABA_B1_) [16, 22, 23], mouse anti-GABA_B1_ monoclonal (sc-166408; D-2, aa 929-958 of rat C-terminus of GABA_B1_, Santa Cruz, CA, USA), guinea pig anti-GIRK2 polyclonal (GP-Af830; aa. 390–421 of mouse GIRK2; Frontier Institute Co., Japan), rabbit anti-GIRK2 polyclonal (Rb-Af290; aa. 390–421 of mouse GIRK2A-1; RRID: AB_2571712; Frontier Institute Co. Japan), rabbit anti-GIRK1 polyclonal (Rb-Af530; aa. 469-501 of mouse GIRK1 C-terminal; RRID: AB_2571711; Frontier Institute Co., Japan), and rabbit anti-GIRK3 polyclonal (Rb-Af750; aa. 358-389 of mouse GIRK3 C-terminal; RRID: AB_2571714; Frontier Institute Co., Japan) antibodies. The characteristics and specificity of the rabbit anti-GABA_B1_ polyclonal antibody have been described elsewhere [32, 33]. The characteristics and specificity of the mouse anti-GABA_B1_ monoclonal antibody have been previously described [9]. The characteristics and specificity of the anti-GIRK1, anti-GIRK2, and anti-GIRK3 antibodies, including SDS-FRL, have been described elsewhere [17, 27, 34].

The secondary antibodies used were as follows: anti-guinea pig IgG conjugated to 10-nm gold particles, anti-guinea pig IgG conjugated to 5-nm gold particles, and anti-rabbit IgG conjugated to 10-nm gold particles (1:100; British Biocell International, Cardiff, UK).

### SDS-digested freeze-fracture replica labelling (SDS-FRL) technique

SDS-FRL was performed with some modifications to the original method described previously [35]. Briefly, hippocampal slices containing the CA1 region were trimmed and immersed in graded glycerol of 10–30% in 0.1 M PB at 4°C overnight. Slices were frozen using a high-pressure freezing machine (HPM010, BAL-TEC, Balzers). Slices were then fractured into two parts at −120°C and replicated by carbon deposition (5 nm thick), platinum (60° unidirectional from horizontal level, 2 nm), and carbon (15–20 nm) in a freeze-fracture replica machine (BAF060, BAL-TEC, Balzers). Replicas were transferred to 2.5% SDS and 20% sucrose in 15 mM Tris buffer (pH 8.3) for 18 h at 80°C with shaking to dissolve tissue debris. The replicas were washed three times in 50 mM Tris-buffered saline (TBS, pH 7.4), containing 0.05% bovine serum albumin (BSA), and then blocked with 5% BSA in the washing buffer for 1 h at room temperature. Next, the replicas were washed and reacted with a polyclonal rabbit antibody for GABA_B1_ (5μg/ml) at 15°C overnight. Following three washes in 0.05% BSA in TBS and blocking in 5% BSA/TBS, replicas were incubated in secondary antibodies conjugated with 10-nm gold particles overnight at room temperature. When the primary antibody was omitted, no immunoreactivity was observed. After immunogold labelling, the replicas were immediately rinsed three times with 0.05% BSA in TBS, washed twice with distilled water, and picked up onto grids coated with pioloform (Agar Scientific, Stansted, Essex, UK).

### Quantification and analysis of SDS-FRL data

The labelled replicas were examined using a transmission electron microscope (JEOL JEM-1400Flash) and photographed with a high-sensitivity sCMOS camera at magnifications of 30,000×. All antibodies used in this study were visualized by immunoparticles on the protoplasmic face (P-face), consistent with the intracellular location of their epitopes. Non-specific background labelling was measured on E-face surfaces in wild type mice. Digitized images were then modified for brightness and contrast using Adobe PhotoShop CS6 (Mountain View, CA, USA) to optimize them for quantitative analysis. The quantitative analyses were done using the software GPDQ (*Gold Particle Detection and Quantification*) developed recently to perform automated and semi-automated detection of gold particles present in each compartment of neurons [27].

#### Density of GIRK subunits along the membrane surface

The procedure was similar to that used previously [27]. Briefly, immunogold labelling for GIRK1, GIRK2, and GIRK3 was achieved from replicas containing the *stratum radiatum* of the CA1 region. Quantitative analysis of immunogold labelling for the GIRK channel subunits was performed on 2 different dendritic compartments of CA1 pyramidal cells and in axon terminals establishing synaptic contacts with spines of pyramidal cells. The dendritic compartments analysed were the spiny branchlets (oblique dendrites) and dendritic spines. Oblique dendrites were identified based on their small diameter and the presence of at least one emerging spine from the dendritic shaft. Spines were considered as such if (i) they emerged from a dendritic shaft or (ii) they opposed an axon terminal. Axon terminals were identified based on (i) the concave shape of the P-face and the accumulation of intramembrane particles (IMPs) on the opposing exoplasmic-face (E-face) of a spine or dendrite or (ii) the presence of synaptic vesicles on their cross-fractured portions. Non-specific background labelling was measured on E-face structures surrounding the measured P-faces. Images of the identified compartments were selected randomly over the *stratum radiatum* of CA1 pyramidal cells and then captured with a high-sensitivity sCMOS camera (JEOL). The area of the selected profiles and the number of immunoparticles were measured using our GPDQ software [27]. Immunoparticle densities were presented as mean ± SEM between animals. Statistical comparisons were performed with GraphPad Prism 5 software (La Jolla, CA, USA).

#### Analysis of the spatial relationship between GIRK channel subunits

Nearest neighbour distances (NNDs) between the 5-nm gold particles (GIRK2) and the 10-nm immunoparticles (GIRK1 or GIRK3) were measured at post- and presynaptic compartments using the GPDQ software. Distances between the two immunoparticles were plotted in bins of 40-nm membrane segments of the plasma membrane. The data expressed in this way show the proximity between two proteins in physiological and pathological conditions.

#### Nanoscale organization of GABA_B1_ receptors and GIRK2 channels

At post- and presynaptic sites, nearest neighbour distances (NNDs) between the 10-nm gold particles (GABA_B1_) and the 5-nm immunoparticles (GIRK2) were measured using the GPDQ software [27]. NNDs between the two kinds of immunoparticles were then compared with those between the real GABA_B1_ and randomly generated GIRK2 particles. Fitted random simulations [27] were generated by first counting the number of GIRK2 particles in each profile. Then, the same number of particles was located randomly within the same delineated area of the dendritic spine. The simulations were considered as valid when there was no statistical difference (Kolmogorov–Smirnov with significance=0.95) between the distributions of all-pair distances in real and simulated GIRK2 particles. If simulations were rejected, then particles were redistributed by means of an iterative algorithm until the condition holds.

### Proximity ligation in situ assay

Proximity ligation in situ assay (P-LISA), using the Duolink detection kit (Olink Bioscience, Uppsala, Sweden), was performed as described elsewhere [36]. Briefly, sections were washed three times in PBS and incubated with blocking solution [10% normal donkey serum (NDS) in PBS; Jackson ImmunoResearch Laboratories, Inc., West Grove, PA, USA] for 2 h at room temperature. Next, the endogenous mouse immunoglobulins were blocked using 100 μg/ml unconjugated Fab fragments anti-mouse IgG (H+L) in TBS for 1 h at room temperature. Subsequently, the sections were incubated with the primary antibodies (mouse anti-GABA_B1_ at 2 μg/ml and rabbit anti-GIRK2 at 1 μg/ml) overnight at 4°C. After two rinses (10 min each) with 1% NDS in PBS, the Duolink detection kit manufacturer’s protocol was followed. To quench lipofuscin autofluorescence, the sections were incubated in 0.1% Sudan black for 30 min. Finally, the sections were washed two times with PBS and mounted on slides with Everbrite hard-set mounting medium (VWR Biotium) containing DAPI. Next, fluorescence images were acquired using a Zeiss LSM800 confocal scanning laser microscope, using a 40×/1.3 oil objective from the *stratum radiatum* of the CA1 region in the hippocampus. High-resolution images were acquired as a Z-stack with a 0.43-μm Z-interval with a total thickness of 10.35 μm. Image processing was performed using the ImageJ Software (NIH) as described elsewhere [37, 38] and particles larger than 0.15 μm^2^ for the P-LISA signal were counted.

### Controls

To test method specificity in the procedures for SDS-FRL, the primary antibody was either omitted or replaced with 5% (v/v) normal serum of the species of the primary antibody, resulting in total loss of the signal. To test for any cross-reactivity of secondary antibodies when double labelling was used by the SDS-FRL technique, some replicas were incubated with only one primary antibody and the full complement of the secondary antibodies. No cross-labelling was detected that would influence the results. In addition, some replicas were incubated with the two primary antibodies, but the size of immunogold in the secondary antibodies for the two target proteins was swapped. No differences in distances of the two target proteins were detected that would influence our results. Finally, when double labelling was used, some replicas were incubated with a cocktail of two primary antibodies (GIRK2 and GIRK1, GIRK2 and GIRK3, or GIRK2 and GABA_B1_) followed by a cocktail of secondary antibodies. Other replicas were incubated with a primary antibody, and then incubated with the second primary antibody, followed by secondary antibodies, and other replicas were incubated with a changed sequence of primary antibodies, applying the first primary antibody for GIRK2 followed by the secondary antibody, and then we applied the second primary antibody (GIRK1, GIRK3, or GABA_B1_) followed by secondary antibody. Under these conditions, we observed similar spatial distribution between two particles; hence, that steric hindrance does not seem to affect interparticle distance. For P-LISA experiments, the standard negative control was the omission of one of the primary antibodies or excluding the P-LISA probes. The use of such controls always yielded no PLA reaction.

### Data analysis

Statistical analyses for morphological data were performed using SigmaPlot 14.5 (Systat, Inpixon, CA, USA) and data were presented as mean ± SEM unless indicated otherwise. Statistical significance was defined as *p* < 0.05. The statistical evaluation of the immunogold densities was performed using the Shapiro–Wilk normality test and Kolmogorov–Smirnov test for the study of normal distribution and an unpaired *t*-test for the comparison of variances. If normal distribution or variances were significatively different, the samples were considered as non-parametric and analysed by the Mann–Whitney test; otherwise, they were considered parametric and analysed by an unpaired *t*-test.

## Results

### Reduction of plasma membrane GIRK2 on the hippocampus of aged APP/PS1 mice

**Fig. 1:**
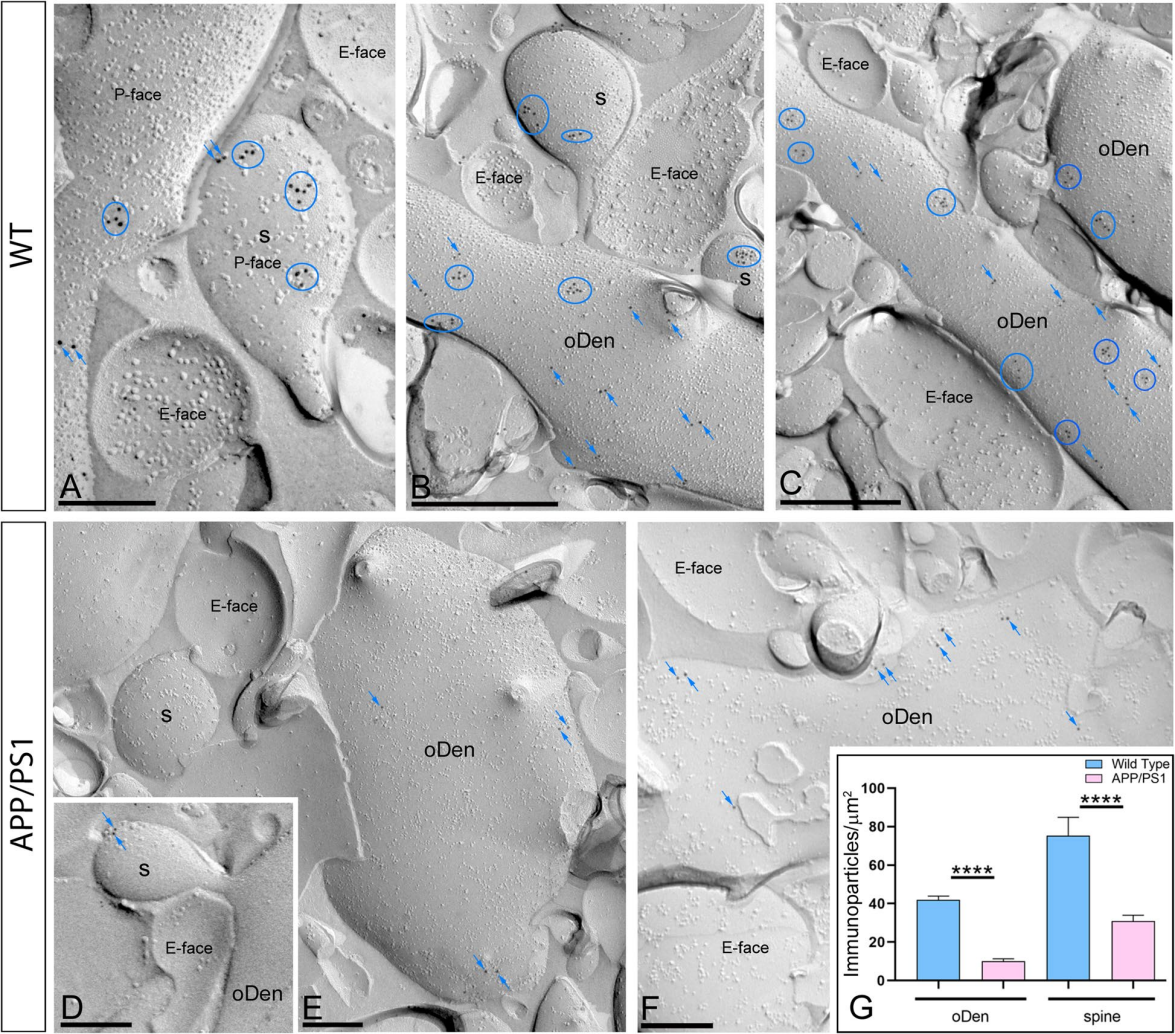
Postsynaptic reduction of GIRK2 in CA1 pyramidal cells of APP/PS1 mice. Electron micrographs showing postsynaptic immunoparticles for GIRK2 in the *stratum radiatum* of the hippocampal CA1 field at 12 months of age, as detected using the SDS-FRL technique in wild type and APP/PS1 mice. **A**–**C** In wild type, GIRK2 immunoparticles were detected forming clusters (blue circles) or scattered (blue arrows) and associated with the P-face in dendritic spines (s) and oblique dendrites (oDen) of CA1 pyramidal cells. **D**–**F** In APP/PS1, very low frequency of clusters or scattered (blue arrows) GIRK2 immunoparticles were observed in dendritic spines (s) and oblique dendrites (oDen) of CA1 pyramidal cells. The E-face is free of any immunolabelling. **G** Quantitative analysis showing that the density of surface GIRK2 immunoparticles was significantly reduced in the APP/PS1 mice compared to age-matched wild type controls in the two subcellular compartments analysed (Mann–Whitney test, ****p < 0.0001). Error bars indicate SEM. Scale bars: **A**–**F**, 0.2 μm

The GIRK2 subunit is an essential component of the functional channel in the hippocampus as it determines both the channel assembly and plasma membrane targeting [14, 39]. Thus, we aimed at investigating whether Aβ pathology affects the spatial organization of the GIRK2 subunit. Hippocampal sections from 12-month-old wild type and APP/PS1 mice were processed for SDS-FRL [35] and the GIRK2 subcellular distribution in pyramidal neurons from the distal part of the *stratum radiatum* of the CA1 region was assessed (Fig. 1). In wild type animals, immunoparticles for GIRK2 were detected in postsynaptic elements, namely, at the extrasynaptic plasma membrane of spines in contact with axon terminals, which are likely deriving from Schaffer collaterals, and dendritic shafts of CA1 pyramidal cells (Fig. 1A–C), as recently reported by pre-embedding experiments [20]. Importantly, immunoparticles for GIRK2 were found mostly in clusters containing 3 or more particles, and in a lesser extent scattered as single gold particles outside the clusters (Fig. 1A–C). Conversely, a reduced density of GIRK2 immunoparticles and fewer channel clusters was found along the membrane surface of CA1 pyramidal cells from APP/PS1 mice (Fig. 1D–F). No labelling was observed on the E-face or on cross-fractures (Fig. 1A–F). Indeed, the quantitative analysis of the images demonstrated that GIRK2 density was significantly reduced in oblique dendrites (oDen) and spines in APP/PS1 mice (oDen = 10.02 ± 1.28 immunoparticles/μm^2^; spines= 30.87 ± 3.09 immunoparticles/μm^2^) when compared to age-match wild type mice (oDen= 41.93 ± 2.86 immunoparticles/μm^2^; spines= 75.35 ± 9.53 immunoparticles/μm^2^) (Mann–Whitney test, ****p<0.0001) (Fig. 1G).

**Fig. 2:**
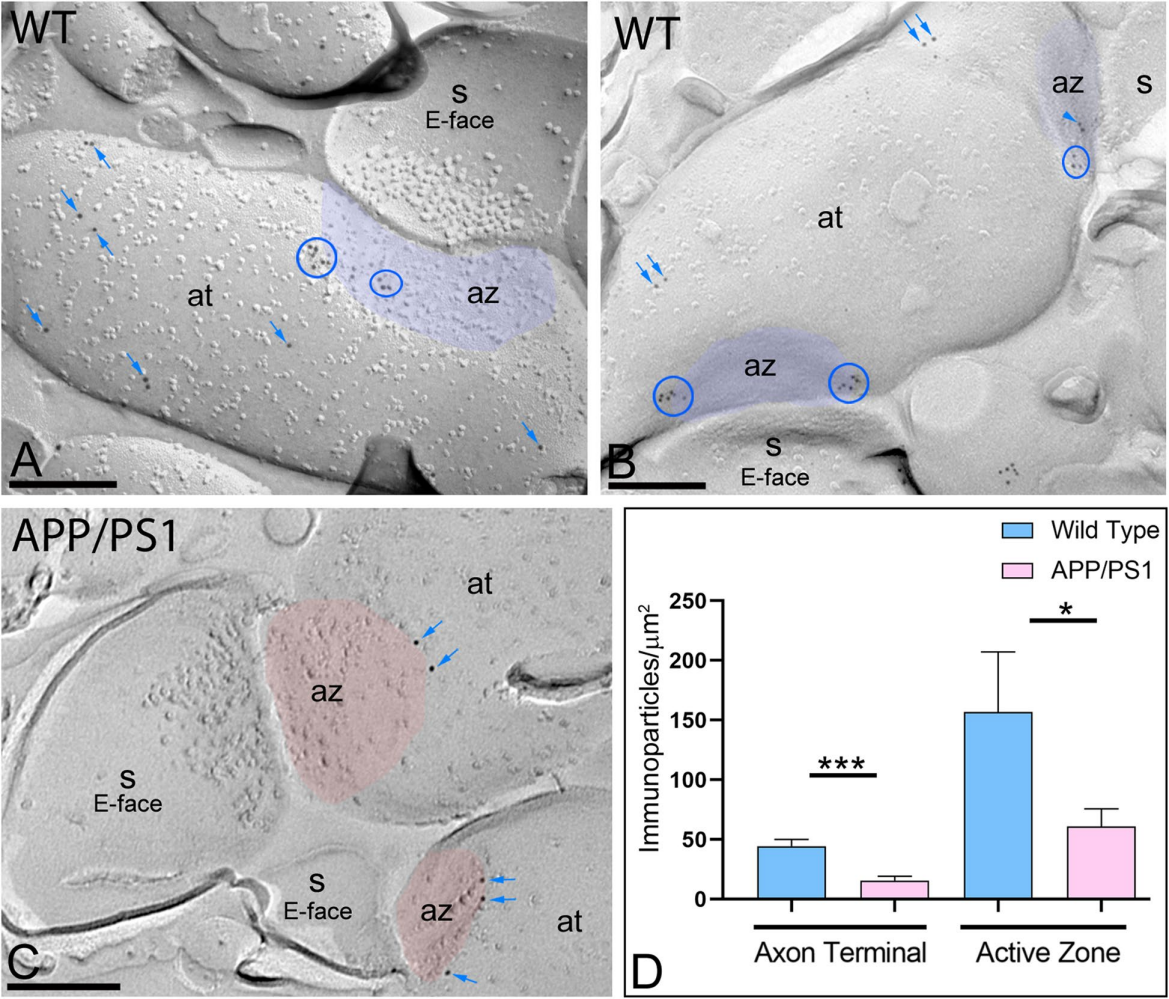
Reduction of GIRK2 immunoparticles in presynaptic compartments of APP/PS1 mice. Electron micrographs showing presynaptic immunoparticles for GIRK2 in the *stratum radiatum* of the hippocampal CA1 field at 12 months of age, as detected using the SDS-FRL technique in wild type and APP/PS1 mice. **A**, **B** In wild type, GIRK2 immunoparticles were mostly found along the extrasynaptic site (blue arrows) of axon terminals (at). Although few immunoparticles were detected within the active zone (az, purple overlay), recognized by the concave shape of the P-face and the accumulation of IMPs, many were detected at the border between the active zone and extrasynaptic sites. In these compartments, GIRK2 immunoparticles were observed forming clusters (blue ellipses/circles) and scattered (blue arrows) outside the clusters. **C** In APP/PS1, fewer GIRK2 immunoparticles forming clusters or scattered (blue arrows), were detected at extrasynaptic sites of axon terminals (at), at the edge of the active zone (az), or within the active zone (red overlay). **D** Quantitative analysis showing that the density of GIRK2 immunoparticles was significantly reduced in the APP/PS1 mice compared to age-matched wild type controls in the two presynaptic compartments analysed (Mann–Whitney test, *p < 0.05; ***p<0.001). Error bars indicate SEM. Scale bars: **A**–**D**, 0.2 μm

In addition to somato-dendritic domains of CA1 pyramidal cells, immunoparticles for GIRK2 were also present in axon terminals (Fig. 2), as previously reported [16, 20]. In wild type mice, GIRK2 immunoparticles were mainly detected at extrasynaptic sites and the immediate perisynaptic region of the active zone and, to a lesser extent, within the active zone, as identified by the concave shape of the P-face and the accumulation of IMPs (Fig. 2A, B). GIRK2 immunoparticles were detected forming clusters or scattered at extrasynaptic sites, the active zone, and the edge of the active zone (Fig. 2A, B). In APP/PS1 mice, GIRK2 immunoparticles were found in the same subcellular compartments as in wild type, but less frequently detected along the surface (Fig. 2C). Our quantitative analysis showed that the density of GIRK2 in presynaptic nerve terminals was significantly reduced in extrasynaptic and the active zone in APP/PS1 mice (extra = 15.66 ± 3.70 immunoparticles/μm^2^; AZ= 61.04 ± 14.69 immunoparticles/μm^2^) compared to age-matched wild type mice (extra = 44.38 ± 5.80 immunoparticles/μm^2^; AZ= 156.82 ± 50.41 immunoparticles/μm^2^) (Mann–Whitney test, *p < 0.05; ***p < 0.001) (Fig. 2D).

### GIRK2 differentially co-clusters with other GIRK channel subunits

**Fig. 3:**
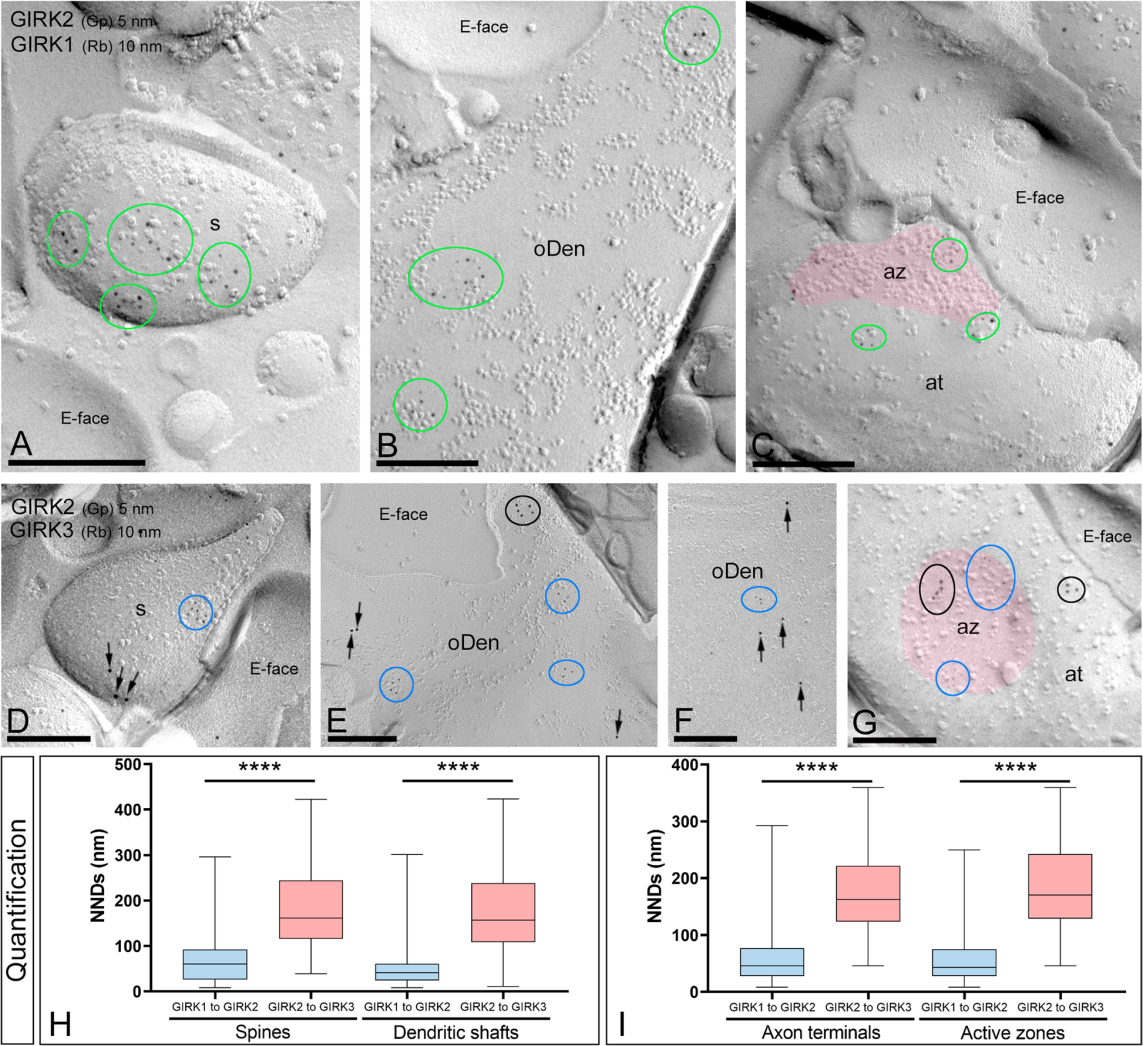
Differential co-clustering of GIRK2 with other GIRK subunits in CA1 pyramidal cells. Electron micrographs of the *stratum radiatum* of the hippocampal CA1 field showing double labelling for GIRK2 (5 nm)/GIRK1 (10 nm) and GIRK2 (5 nm)/GIRK3 (10 nm), as detected using the SDS-FRL technique. **A**–**C** Immunoparticles for GIRK2 co-clustered with those for GIRK1 (green ellipses/circles) in dendritic spines (s), oblique dendrites (oDen), and axon terminals (at). **D**–**G** Immunoparticles for GIRK2 (blue ellipses/circles) were segregated from GIRK3 clusters (black ellipses/circles) or immunoparticles (black arrows) in dendritic spines (s), oblique dendrites (oDen), and axon terminals (at). **H** Quantitative analysis using the SDS-FRL technique, showing the nearest neighbour distance (NND) between immunoparticles for GIRK2 to GIRK1 and immunoparticles for GIRK2 to GIRK3 in spines and dendritic shafts, as well as presynaptically in axon terminals and their active zones. The distances between immunoparticles for GIRK2 and GIRK1 are significantly shorter in all neuronal compartments than those between GIRK2 and GIRK3 (Mann–Whitney test, ****p < 0.0001). Thus, this analysis demonstrated a spatial association between GIRK2 and GIRK1, but not between GIRK2 and GIRK3. Scale bars: **A**–**G**, 0.2 μm

It is well known that GIRK2 may assemble with other GIRK subunits to form heteromeric channels [2]. Thus, we assessed GIRK2 co-clustering with other GIRK subunits by double-labelling SDS-FRL experiments. First, we provided morphological insights into the GIRK1-GIRK2 association (Fig. 3A–C). Immunoparticles for GIRK2 co-clustered with those for GIRK1 in all neuronal compartments including the extrasynaptic plasma membrane of spines, dendritic shafts, and axon terminals (Fig. 3A–C). Next, we performed double labelling to investigate the spatial relationship between GIRK2 and GIRK3 (Fig. 3D–G). Although many immunoparticles for GIRK2 were distributed close to GIRK3 immunoparticles, most of them were not co-clustering in spines, dendritic shafts, or axon terminals (Fig. 3D–G).

To quantitatively assess the extent of the spatial relationship between GIRK channel subunits, we measured the nearest neighbour distances (NNDs) between the 5-nm gold particles (GIRK2) and the 10-nm immunoparticles (GIRK1 or GIRK3). The medians of the NNDs between GIRK1 and GIRK2 immunoparticles were 60.6 nm (interquartile range, 25.8–92.2 nm) in spines, 41.5 nm (interquartile range, 24.19–60.62 nm) in dendritic shafts, 45.7 nm (interquartile range, 27.2–76.7 nm) in extrasynaptic axon terminals, and 43.1 nm (interquartile range, 27.5–75.1 nm) in the active zone of axon terminals (Fig. 3H). The medians of the NNDs between GIRK3 and GIRK2 immunoparticles were 161.6 nm (interquartile range, 115.7–244.3 nm) in spines, 156.5 nm (interquartile range, 108.5–237.8 nm) in dendritic shafts, 162.2 nm (interquartile range, 123.3–221.9 nm) in extrasynaptic axon terminals, and 170.4 nm (interquartile range, 128.8–242.6 nm) in the active zone of axon terminals (Fig. 3I). These distances were significantly shorter in all neuronal compartments for GIRK2 and GIRK1 than those for GIRK2 and GIRK3 (Mann–Whitney test, ****p < 0.0001). Overall, these results suggest that GIRK1 but not GIRK3 subunits are specifically targeted to have a close association with GIRK2 channels both at postsynaptic and presynaptic compartments.

### Reduction of GIRK1 density, but not GIRK3, in aged APP/PS1 mice

**Fig. 4:**
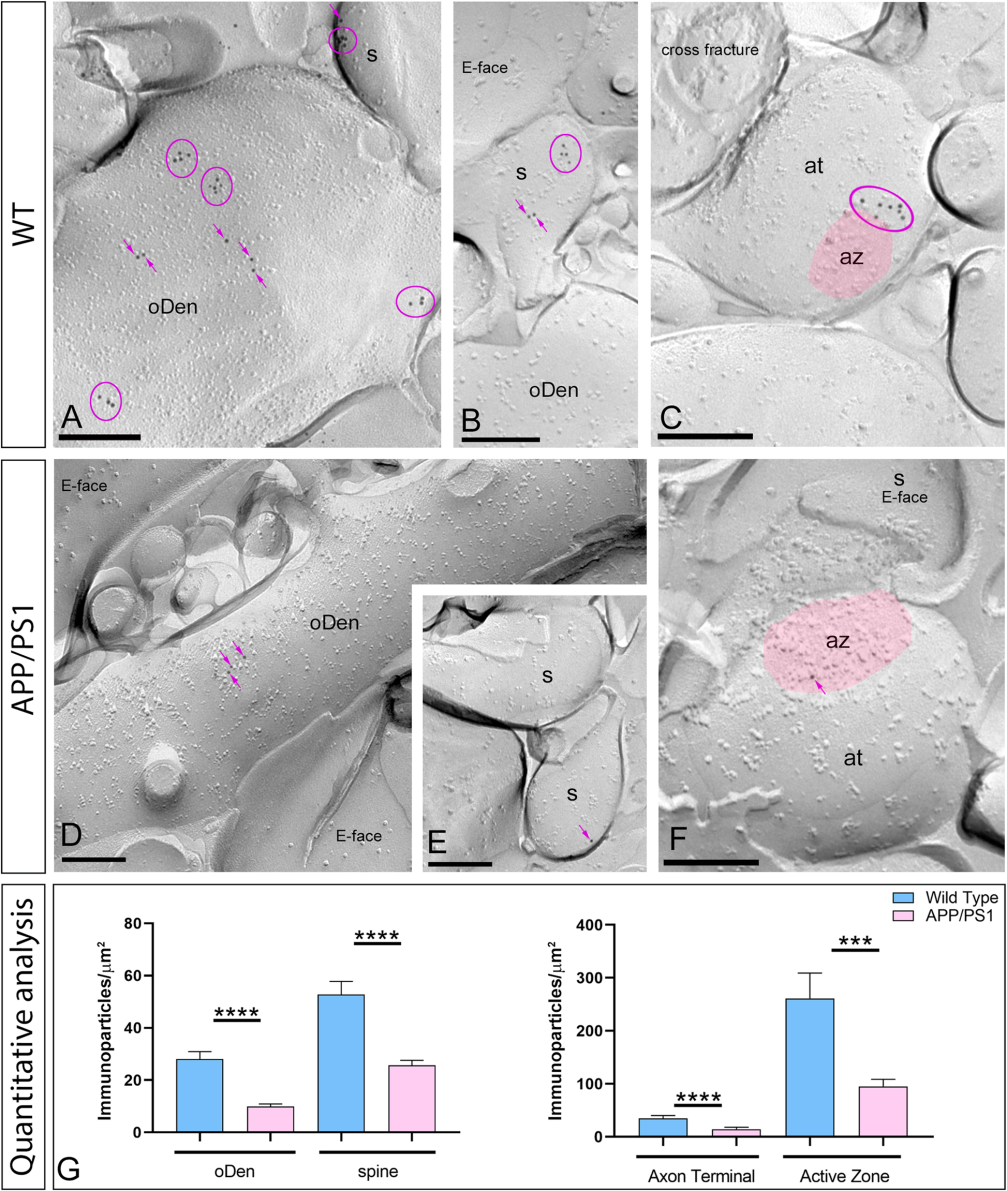
Reduction of GIRK1 immunoparticles in post- and presynaptic compartments of APP/PS1 mice. Electron micrographs showing immunoparticles for GIRK1 in the *stratum radiatum* of the hippocampal CA1 field at 12 months of age, as detected using the SDS-FRL technique in wild type and APP/PS1 mice. **A**–**C** In wild type, immunoparticles for GIRK1 were detected forming clusters (pink circles) or scattered (pink arrows) in dendritic spines (s) and oblique dendrites (oDen) of CA1 pyramidal cells, as well as presynaptically along the extrasynaptic site and in the active zone (az, pink overlay) of axon terminals (at). **D**–**F** In APP/PS1, very low frequency of clusters or scattered (purple arrows) GIRK1 immunoparticles were observed in dendritic spines (s), oblique dendrites (oDen), or axon terminals (at). The E-face is free of any immunolabelling. **G** Quantitative analysis showing that the density of GIRK1 immunoparticles was significantly reduced in the APP/PS1 mice compared to age-matched wild type controls in the post- and presynaptic subcellular compartments analysed (Mann–Whitney test, ***p < 0.001; ****p<0.0001). Error bars indicate SEM. Scale bars: **A**–**F**, 0.2 μm

Since GIRK1 and GIRK3 are differentially associated to GIRK2, we next interrogated whether Aβ pathology also affects differently to the spatial organization of these subunits. Thus, the nanoscale organization of GIRK1 and GIRK3 in different compartments of CA1 pyramidal cells was assessed as described above. In wild type mice, immunoparticles for GIRK1 were mostly distributed at the extrasynaptic plasma membrane of spines and dendritic shafts of CA1 pyramidal cells, either forming clusters or scattered as single gold particles outside the clusters (Fig. 4A, B). GIRK1 immunoparticles were also detected along the extrasynaptic site of axon terminals or around the edge of the active zone of axon terminals, and less frequently within the active zone (Fig. 4C). Conversely, in APP/PS1 mice, few immunoparticles for GIRK1 were detected in clusters or scattered along the membrane surface of spines and dendritic shafts of CA1 pyramidal cells or at presynaptic sites (Fig. 4D–F). No labelling was observed on the E-face or on cross-fractures (Fig. 4A–F). Importantly, the quantitative analysis of the images revealed that GIRK1 density was significantly reduced in oblique dendrites (oDen) and spines in APP/PS1 mice (oDen = 9.94 ± 0.98 immunoparticles/μm^2^, *n*=35 dendrites; spines= 25.72 ± 1.82 immunoparticles/μm^2^, *n*=37 spines) compared to age-match wild type mice (oDen = 28.13 ± 2.79 immunoparticles/μm^2^, *n*=36 dendrites; spines= 52.74 ± 5.05 immunoparticles/μm^2^, *n*=24 spines) (Mann–Whitney test, ****p<0.0001) (Fig. 4G–F). At presynaptic sites, the density of GIRK1 was also significantly reduced at extrasynaptic sites and in the active zone in APP/PS1 mice (extra= 14.26 ± 3.16 immunoparticles/μm^2^, *n* = 24 extrasynaptic sites; AZ = 95.12 ± 13.41 immunoparticles/μm^2^, *n*= 22 active zones) compared to age-match wild type mice (extra= 36.03 ± 4.36 immunoparticles/μm^2^, *n* = 29 extrasynaptic sites; AZ = 261.30 ± 47.74 immunoparticles/μm^2^, *n*= 19 active zones) (Mann–Whitney test, ***p<0.001; ****p< 0.0001) (Fig. 4). The densities of immunoparticles for GIRK1 on the three compartments were significantly different (Mann–Whitney test, *p* < 0.001) from the background labelling determined on the surrounding E-face plasma membranes (0.91 ± 0.08 immunoparticles/μm^2^).

**Fig. 5:**
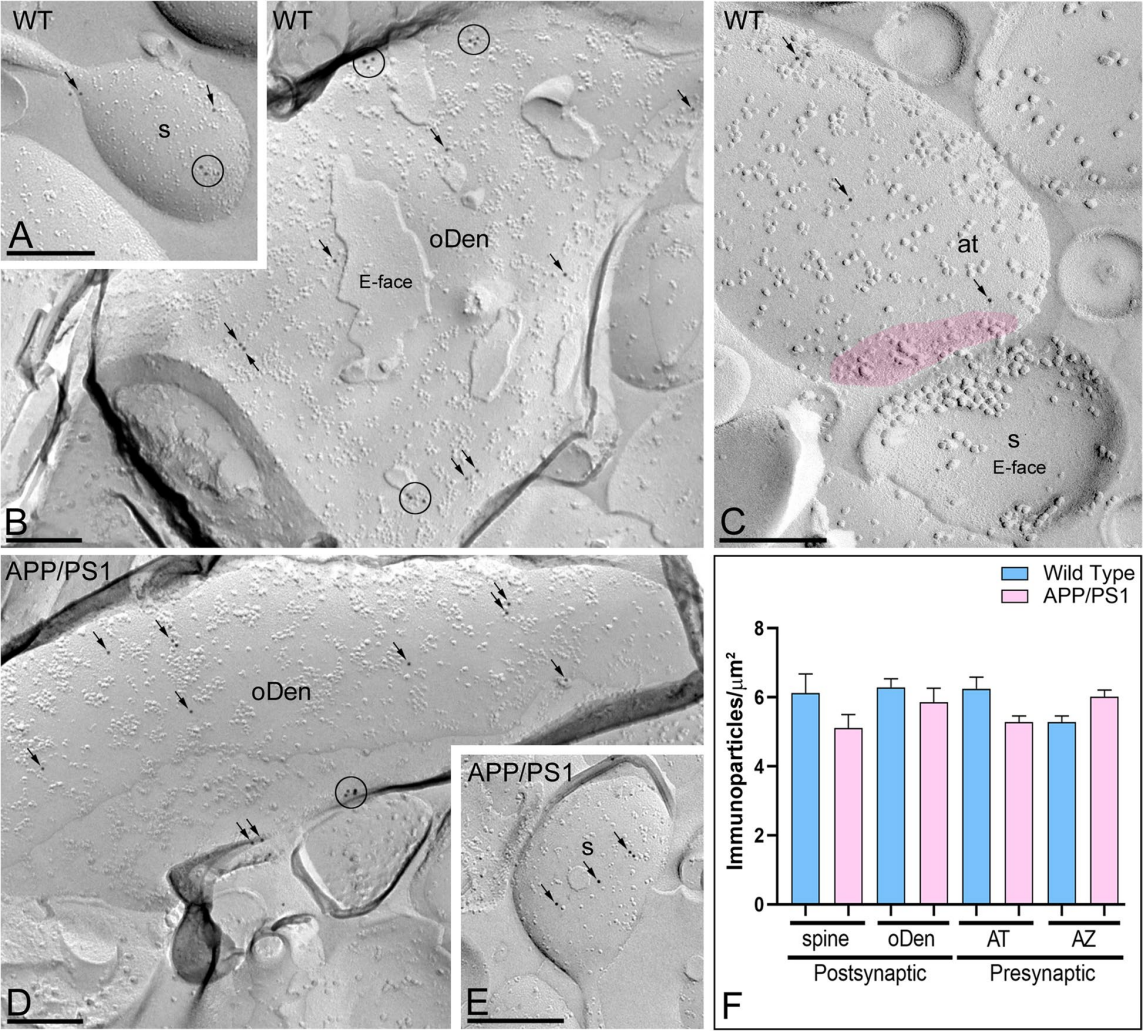
Unaltered distribution of GIRK3 in CA1 pyramidal cells of APP/PS1 mice. Electron micrographs showing immunoparticles for GIRK3 in the *stratum radiatum* of the hippocampal CA1 field at 12 months of age, as detected using the SDS-FRL technique in wild type and APP/PS1 mice. **A**–**E** Both in wild type and APP/PS1, immunoparticles for GIRK3 were detected mostly scattered **(**black arrows), or less frequently forming clusters (black circles), in dendritic spines (s) and oblique dendrites (oDen) of CA1 pyramidal cells, as well as presynaptically along the extrasynaptic site and active zone (az, pink overlay) of axon terminals (at). **F** Quantitative analysis showing that the density of GIRK3 immunoparticles was unaltered in wild type and APP/PS1 mice in the post- and presynaptic subcellular compartments analysed (Mann–Whitney test, *p* = 0.14 for spines, *p* = 0.35 for dendritic spines, *p* = 0.064 for extrasynaptic sites, *p* = 0.369 for active zone sites). Error bars indicate SEM. Scale bars: **A**–**F**, 0.2 μm

In contrast to GIRK2 and GIRK1, fewer immunoparticles for GIRK3 were detected in postsynaptic and presynaptic compartments, thus showing mostly a scattered distribution as opposed to forming clusters, both in wild type and APP/PS1 mice (Fig. 5A–E). In addition, the subcellular distribution pattern and GIRK3 density observed in wild type (Fig. 5A–C) were similar to those found in APP/PS1 mice (Fig. 5D, E). Quantitative comparison of the GIRK3 densities along the neuronal surface of CA1 pyramidal cells in the *stratum radiatum* revealed two main findings: (1) a uniform density of GIRK3 immunoparticles in spines, dendritic shafts, and axon terminals and (2) similar low GIRK3 densities along in wild type and APP/PS1 mice (Fig. 5F). Indeed, our analysis demonstrated that GIRK3 density did not change in APP/PS1 mice (oDen = 5.11 ± 0.38 immunoparticles/μm^2^, *n*=36 dendrites; spines= 5.86 ± 0.41 immunoparticles/μm^2^, *n*=36 spines; extra = 5.18 ± 1.19 immunoparticles/μm^2^, *n* = 27 terminals; AZ= 4.29 ± 0.59 immunoparticles/μm^2^, *n*= 24 terminals) compared to age-match wild type mice (oDen = 6.12 ± 0.55 immunoparticles/μm^2^, *n*=36 dendrites; spines= 6.29 ± 0.24 immunoparticles/μm^2^, *n*=36 spines; extra= 6.24 ± 0.36 immunoparticles/μm^2^, *n* = 27 terminals; AZ = 4.63 ± 0.62 immunoparticles/μm^2^, *n*= 27 terminals) (Mann–Whitney test, *p* = 0.14 for spines, *p* = 0.35 for dendritic spines, *p* = 0.064 for extrasynaptic sites, *p* = 0.369 for active zone sites) (Fig. 5). These density values in all examined compartments were above the non-specific labelling determined on the surrounding E-face plasma membranes (background: 0.98 ± 0.09 immunoparticles/μm^2^; Mann–Whitney test, *p* < 0.01).

In summary, SDS-FRL labelling for GIRK1 and GIRK3 subunits showed clear subunit- and compartment-specific differences. While GIRK1 displayed higher densities with differences along the dendritic axis of CA1 pyramidal cells, mirroring GIRK2 distribution, GIRK3 had lower density of immunolabelling and was uniform along the axo-dendritic axis.

### Reduced interaction of GABA_B_ and GIRK2 on the hippocampus of aged APP/PS1 mice

**Fig. 6:**
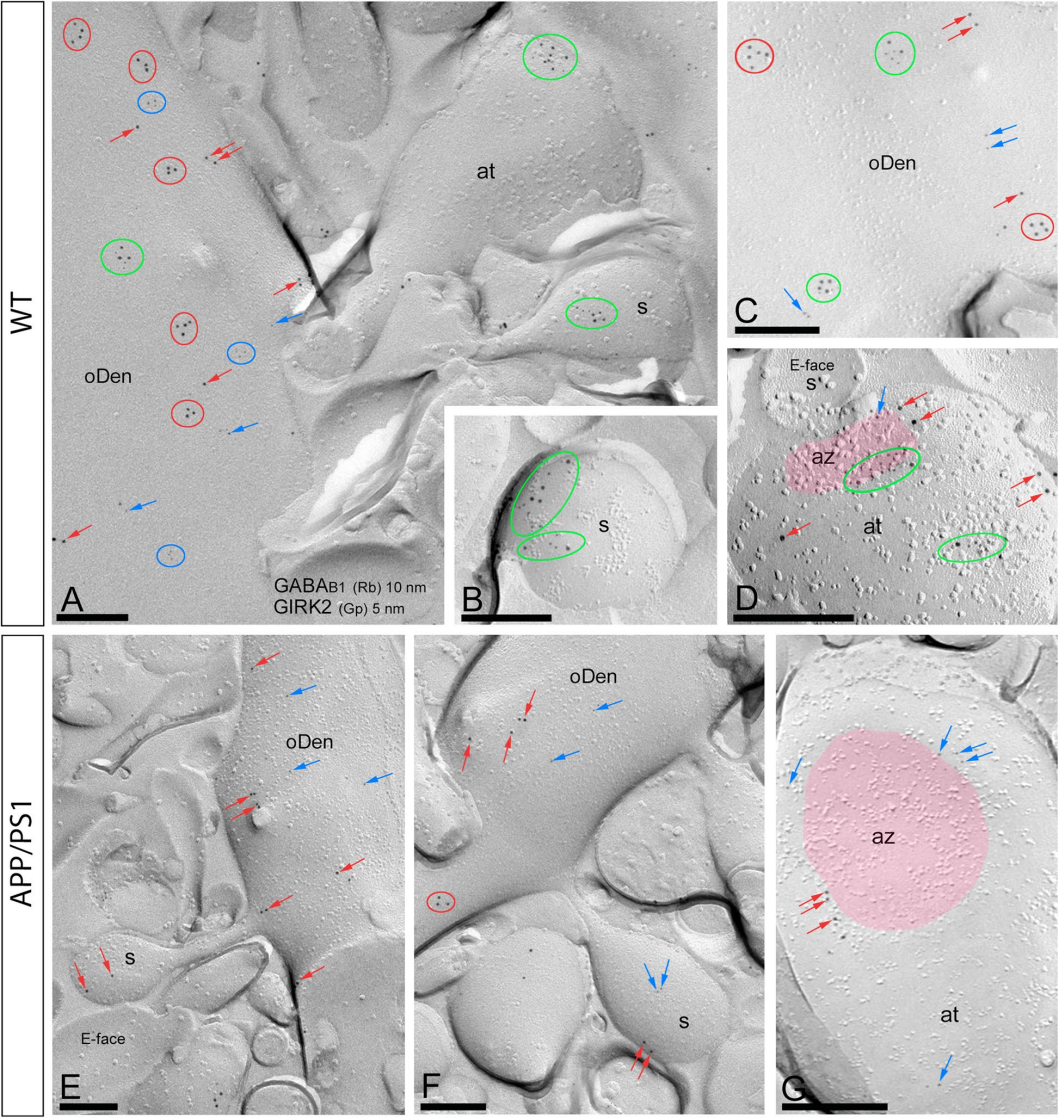
Co-clustering of GABA_B_ receptors and GIRK2 in the hippocampus of wild type and APP/PS1. Electron micrographs of the *stratum radiatum* of the hippocampal CA1 field at 12 months of age showing double immunogold labelling for GABA_B1_ (10 nm) and GIRK2 (5 nm) in pyramidal cells, as detected using the SDS-FRL technique in wild type and APP/PS1 mice. **A**–**D** In wild type, immunoparticles for GABA_B1_ (10 nm) co-clustered with those for GIRK2 (5 nm) (green ellipses/circles) in dendritic spines (s). In oblique dendrites (oDen), double labelling revealed that many clusters (red ellipses/circles) and immunoparticles (red arrows) for GABA_B1_ (10 nm) were segregated, and also clusters (blue ellipses/circles) and immunoparticles (blue arrows) for GIRK2 (5 nm) could be found, although in some cases clusters of the two proteins (green ellipses/circles) were also detected. Presynaptically, immunoparticles for GABA_B1_ (10 nm) co-clustered (green ellipses/circles) with those for GIRK2 (5 nm) in axon terminals (at) and edge of active zone (az, pink overlay). **E**–**G** In APP/PS1, immunoparticles for GABA_B1_ (10 nm, red arrows) were segregated from immunoparticles for GIRK2 (*5* nm, blue arrows) in spines (s), oblique dendrites (oDen), or axon terminals (at). Scale bars: **A**–**G**, 0.2 μm

The functional and molecular coupling of GABA_B_ receptors and GIRK channels in the hippocampus has been previously reported [16, 40]. Since a dysfunction on GABA_B_-GIRK signalling in AD has been postulated [9–11], we interrogated whether the molecular interaction would also be altered. To this end, we first demonstrated that the density of GABA_B_ receptors was significantly reduced in spines and oblique dendrites (oDen) in APP/PS1 mice (s = 43.87 ± 7.83 immunoparticles/μm^2^, *n*=16 spines; oDen= 32.12 ± 2.32 immunoparticles/μm^2^, *n*=61 dendrites) compared to age-matched wild type mice (s = 94.39 ± 8.155 immunoparticles/μm^2^, *n*=32 spines; oDen = 86.25 ± 2.88 immunoparticles/μm^2^, *n*=22 dendrites) (Mann–Whitney test, *p*<0.001). The data confirmed that the membrane localization of GABA_B_ receptors was altered in APP/PS1 mice, similarly to GIRK1 and GIRK2. Next, we assessed the GABA_B_-GIRK association on the hippocampus of aged APP/PS1 mice through double-labelling SDS-FRL and P-LISA. Due to the fact that our anti-GIRK1 and anti-GIRK3 antibodies were raised in the same species as our anti-GABA_B1_ antibody, these experiments were conducted only with the anti-GIRK2 antibody. Firstly, double-labelling SDS-FRL experiments in wild type animals revealed that GABA_B1_ co-clustered with GIRK2 along the extrasynaptic plasma membrane of dendritic spines (Fig. 6A, B). In dendritic shafts, clusters of GABA_B1_ immunoparticles appeared to be mostly segregated from those of GIRK2 (Fig. 6A). However, a low degree of co-clustering of the immunoparticles for the two proteins was also observed (Fig. 6A, C). At presynaptic sites, the channels and receptors were mainly co-clustering, both at extrasynaptic sites, around the edge of the active zone of axon terminals and within the active zone (Fig. 6D). In APP/PS1 mice, immunoparticles for GABA_B1_ and GIRK2 were mostly detected scattered along the membrane surface of spines and dendritic shafts of CA1 pyramidal cells and axon terminals (Fig. 6E–G). In addition, immunoparticles for GABA_B1_ were always segregated from those for GIRK2 along the extrasynaptic plasma membrane of spines, dendritic shafts, or axon terminals (Fig. 6E–G).

**Fig. 7:**
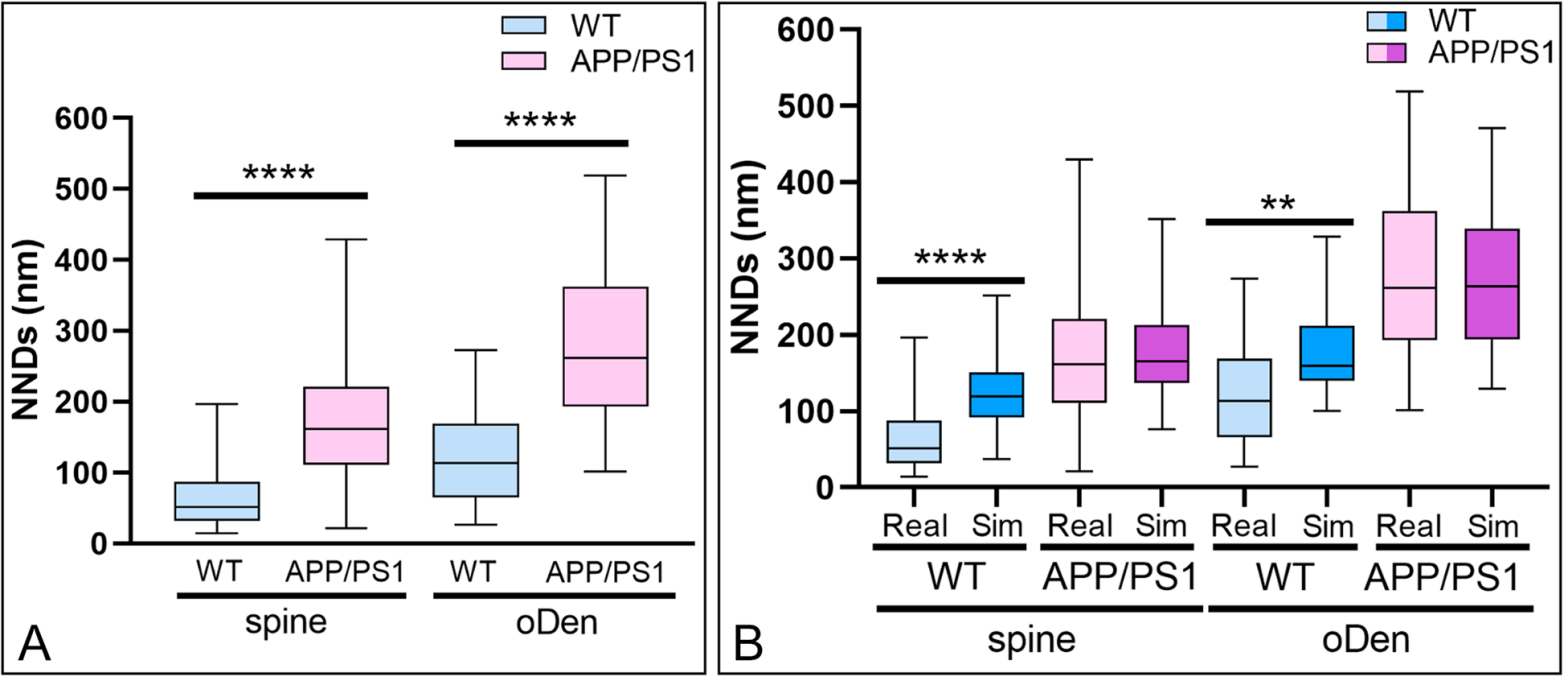
Nanoscale organization of GABA_B_ receptors and GIRK2 channels in the hippocampus of wild type and APP/PS1. **A** Nearest neighbour distances (NNDs) between the 10-nm gold particles (GABA_B1_) and the 5-nm immunoparticles (GIRK2) were measured in spines and oblique dendrites (oDen) using the GPDQ software. The distances (median values) between immunoparticles for GIRK2 and GABA_B1_ are shorter in the wild type than in APP/PS1 mice, both in spines and oblique dendrites (oDen) of CA1 pyramidal cells (****p < 0.0001, Mann–Whitney *U* test). **B** We compared NNDs from real and simulated GIRK2 particles to real GABA_B1_ immunoparticles in dendritic shafts and spines, showing significantly larger NNDs for the randomly distributed GIRK2 immunoparticles compared to the real distributions (**p < 0.01, **** *p* < 0.0001, Mann–Whitney *U* test)

To examine the extent of the spatial coupling between GABA_B1_ and GIRK2, the NNDs between immunogold particles for GIRK2 (5 nm) with immunogold particles for GABA_B1_ (10 nm) were measured in our double-labelled replicas in wild type and APP/PS1 mice. In dendritic spines, the medians of the NNDs between GIRK2 and GABA_B1_ particles were 51.5 nm (interquartile range, 14.3–196.3 nm) in wild type and 161.5 nm (interquartile range, 21.2–429.2 nm) in APP/PS1 mice (Fig. 7A). In dendritic shafts, the medians of the NNDs between GIRK2 and GABA_B1_ particles were 113.5 nm (interquartile range, 26.9–273.4 nm) in wild type and 261.9 nm (interquartile range, 101.7–518.6 nm) in APP/PS1 mice. These median values were significantly different (****p < 0.0001, Mann–Whitney *U* test) (Fig. 7A). We then conducted fitted simulations of GIRK2 immunoparticles and compared NNDs from real and simulated GIRK2 particles to real GABA_B1_ immunoparticles in dendritic shafts and spines (Fig. 7B). To quantify their extent of spatial relation, the NNDs between immunoparticles for GABA_B1_ and GIRK2 were compared with those between real GABA_B1_ and simulated GIRK2 particles in spines and dendritic shafts (Fig. 7B). We found significantly larger NNDs for the randomly distributed GIRK2 immunoparticles (spines, median, 118.7nm; oDen, median: 159.1 nm) compared to the real distributions (**p < 0.01, **** *p* < 0.0001, Mann–Whitney *U* test, Fig. 7B). Therefore, we found a significant association of GABA_B1_ with GIRK2 in spines and to a lesser extent in the oblique dendrites of the wild type mice while this association was not present in the APP/PS1 mice.

**Fig. 8:**
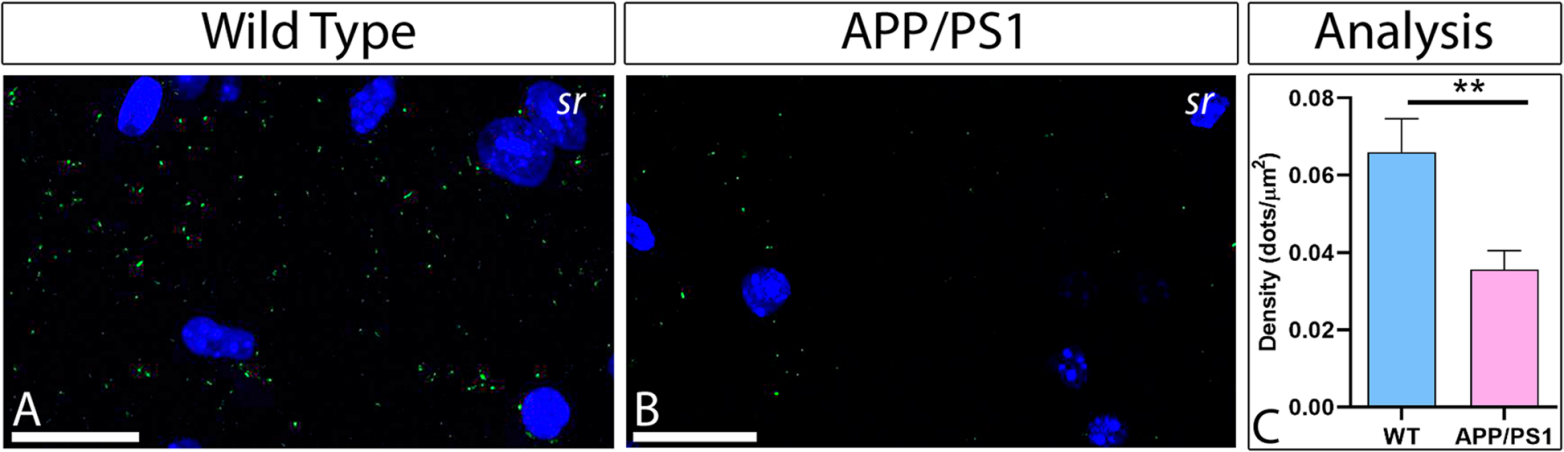
Detection of GABA_B1_-GIRK2 heterodimers in the hippocampus of wild type and APP/PS1. **A**, **B** Photomicrographs of nuclei staining (DAPI) and dual recognition in green of GABA_B1_ and GIRK2 with the proximity ligation in situ assay (P-LISA), in the *stratum radiatum* of CA1 area of WT and APP/PS1 mice. The interaction between GABA_B1_ and GIRK2 is observed by the green dots. **C** Quantification of P-LISA signals for GABA_B1_ and GIRK2 proximity confirmed the significant reduction in the density of dots/μm^2^ in APP/PS1 mice compared to age-match wild type (**p<0.01, *t*-test). Values correspond to the mean ± SEM. Scale bars: 20 μm

Subsequently, to sustain the results obtained by quantitative SDS-FRL we performed P-LISA, a method able to identify protein-protein interactions in situ (at distances < 40 nm) [41]. Thus, GABA_B1_ and GIRK2 complexes were detected by P-LISA in the *stratum radiatum* on the hippocampus of wild type and APP/PS1 at 12 months as previously described [37]. P-LISA signal was observed as fluorescence dots representing interactions between GABA_B1_ and GIRK2 (Fig. 8A, B). In wild type, we found that the neuropile of the *stratum radiatum* was decorated with the P-LISA dots (Fig. 8A), whereas a consistent reduction of dots in APP/PS1 hippocampus was observed (Fig. 8B). When evaluating the P-LISA signal using quantitative approaches, the density of fluorescent P-LISA dots was significantly reduced in the hippocampus of APP/PS1 mice when compared to age-matched wild type mice (Fig. 8C) (*p* < 0.01). Overall, our data clearly support the idea that a GABA_B1_ and GIRK2 interaction occurs in the hippocampus and that this interaction is significantly downregulated in an animal model of AD.

## Discussion

In the present study, we report the two-dimensional distribution of GIRK channel subunits and their spatial interplay with GABA_B_ receptors on the hippocampus of APP/PS1 mice. To our knowledge, this is the first-time demonstration of nanoscale alterations in a key hippocampal receptor–effector system in a model of AD. GABA_B_ receptor and GIRK channel nanoscale organization was assessed by quantitative immunogold SDS-FRL technique, and their physical interaction (i.e. oligomerization) further validated through P-LISA experiments. Our data provide compelling evidence demonstrating alterations in GABA_B1_ and GIRK2 density and assembly in a mouse model of AD, thus adding a new piece of information to the AD puzzle.

GIRK channels have been related to AD pathophysiology, yet the evidence was somewhat controversial, likely due to the use of different experimental models of AD. For instance, the incubation of rat hippocampal slices with the Aβo25-35 peptide reduced GIRK mRNA levels [19], whereas the Aβo1-42 did not alter GIRK1 and GIRK2 protein density [42]. Moreover, the oligomeric Aβ42 induced an upregulation of GIRK channel expression and function in the cell surface of neurons [43]. Likewise, animal models of AD have also been a source of disagreement. While no change in total GIRK1 or GIRK2 protein was detected in 12-month-old APP/PS1 mice, significant reductions were found in 10-month-old P301S mice [20]. Nevertheless, we recently reported a 30–40% reduction of GIRK2 along the plasma membrane of hippocampal pyramidal cells from both APP/PS1 and P301S mice [20]. Here, we demonstrated that the density of GIRK2 and GIRK1, but not GIRK3, was significantly reduced along the neuronal surface of CA1 pyramidal cells, as well as in axon terminals contacting these neurons, from 12-month-old APP/PS1 mice. The strikingly parallel reduction in the density of GIRK1 and GIRK2, together with the reduction of GIRK1 labelling observed in the hippocampus of GIRK2 KO mice [16], is compatible with the idea that a significant fraction of hippocampal GIRK channels, both in AD and healthy animals, are heteromultimers composed of GIRK1 and GIRK2. Consistent with this idea, our data show the co-clustering of GIRK1 and GIRK2 in the nanodomain range (~40–50 nm NND) within P-face IMP clusters in spines and shafts of CA1 pyramidal cells. In addition, we provide unequivocal evidence that GIRK2 and GIRK3 do not co-cluster (the NND analysis revealed distances among proteins larger than 156 nm). Overall, our results support the notion that under physiological conditions a significant fraction of hippocampal GIRK channels are heteromultimers composed of GIRK1 and GIRK2, but not GIRK3, and these are downregulated in APP/PS1 mice. The unique hippocampal GIRK3 profile, showing lower density, uniform distribution (i.e. lack of co-clustering), and no apparent alteration in APP/PS1 mice, makes its function intriguing in this brain region and likely is involved in yet undisclosed signalling pathways.

GABA_B_-GIRK currents have been extensively described on the hippocampus [5, 16, 44]. There is experimental evidence suggesting that these currents would be physically maintained by the formation of GPCR–effector macromolecular membrane assemblies (GEMMAs), a concept which has been recently reviewed [24]. Accordingly, it has been postulated that GABA_B_-GIRK MMA will be comprised by some, if not all, of the essential GABA_B_-GIRK signalling complex partners (i.e. GPCR, heterotrimeric G protein and effector ion channels like GIRK), thus enabling a fast and precise signalling. Indeed, the data presented here showing a short nanoscale (~50 nm) distance between the ion channel and the receptor in spines, together with the rapid activation of GIRK channels by GABA_B_ receptors [44, 45] and the existence of pre-assembled GABA_B_ receptor–GIRK channel complexes [40], would support this hypothesis. In addition, our experiments also revealed that a proportion of receptors and ion channels were scattered along the membrane surface, thus not forming GEMMAs. In such scenario, one would expect, if any, a different signalling mode based in collision coupling (i.e. kiss-and-run like). Indeed, both signalling modes (i) GEMMA, granting more efficient and restricted signalling, and (ii) collision coupling, less efficient but more amplified signalling, would likely co-exist in the same CA1 pyramidal cells and balanced according to plasticity requirements. In line with this, our immunogold labelling revealed a high degree of GABA_B1_-GIRK2 co-clustering in spines of CA1 pyramidal cells and presynaptically in axon terminals, precisely where a fast and precise signalling integration is needed. In addition, GABA_B_ receptors and GIRK channels were largely segregated within the dendritic shafts, mostly scattered but also as low but consistent co-clusters. Previous reports using the same immune EM techniques did not report this low dendritic co-clustering in the same neuron population [23]. This discrepancy may be explained by the use of different anti-GIRK2 antibodies, resulting in variations in the efficiency of immunogold labelling between replicas. Our findings, however, are in full agreement with electrophysiological studies reporting that baclofen, a GABA_B_ receptor agonist, evoked GIRK currents only in a subset of isolated patches of CA1 pyramidal cell dendrites [46]. Altogether, our results are indicative that a precise nanoscale organization of GABA_B_ receptors and GIRK channels in CA1 pyramidal cells would be needed to maintain proper receptor–channel signalling and neuronal plasticity.

A hallmark of Alzheimer’s pathology is the misfolding, aggregation, and accumulation of proteins, which leads to cellular dysfunction and synapse loss. In addition, neurotransmitter systems, including the GABAergic, undergo a dynamic remodelling. For instance, in APP/PS1 mice, reactive hippocampal astrocytes produce and secrete the gliotransmitter GABA which activates extrasynaptic GABA receptors and inhibits synaptic function, thus triggering memory and cognitive deficits [47]. Here, we reported that the nanoscale organization of GABA_B_ receptors and GIRK channels is also altered on the hippocampus of the same animal model of AD. Accordingly, the distance between GIRK2 and GABA_B1_ particles increased from 51 to 113 nm in spines and from 161 to 262 nm in dendritic shafts in the mouse model of AD. In addition, a downregulation in GABA_B_–GIRK oligomerization was also demonstrated by P-LISA. Thus, our data is in line with a profound remodelling of GABAergic signalling in the AD brain. In summary, this work explored the pre- and postsynaptic alteration of the three neuronal GIRK channel subunits and their spatial relationship with GABA_B_ receptors in hippocampal CA1 pyramidal cells in the APP/PS1 mouse model of AD. We provide novel evidence for the specific reduction of GIRK1/GIRK2 heteromultimers and for the uncoupling of GIRK channels and GABA_B_ receptors in pyramidal cells on the hippocampus in this mouse model of AD. Finally, the changes in the nanoscale organization of receptors and channels suggest the existence of major molecular rearrangements in APP/PS1 mice, remodelling GABA_B_–GIRK2 interactions, thus urging the design of corrective strategies towards rescuing GABAergic neurotransmission in AD.

## Data Availability

All data used and/or analysed during the current study are available from the corresponding author on reasonable request.
